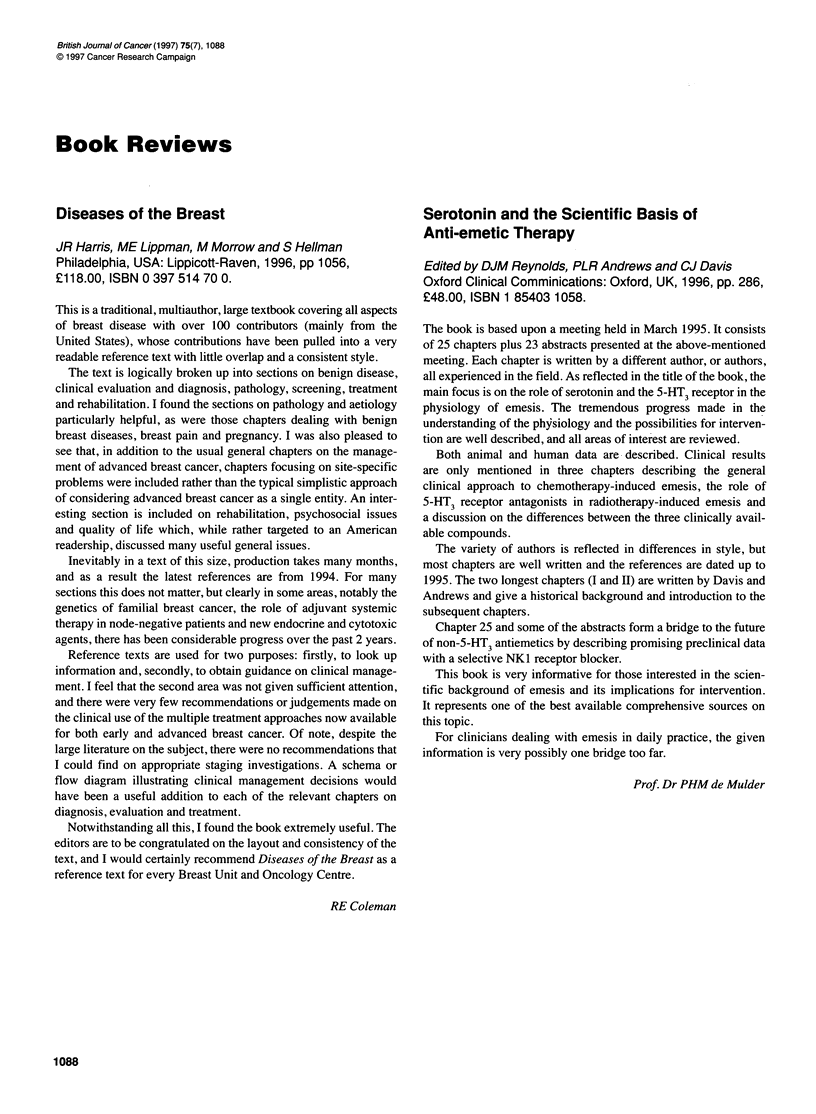# Diseases of the Breast

**Published:** 1997

**Authors:** RE Coleman


© 1997 Cancer Research Campaign

### Book Reviews

### Diseases of the Breast

*JR Harris, ME Lippman, M Morrow and S Hellman*
Philadelphia, USA: Lippicott-Raven, 1996, pp 1056, £118.00, ISBN 0 397 514 70 0.

This is a traditional, multiauthor, large textbook covering all aspects of breast disease with over 100 contributors (mainly from the United States), whose contributions have been pulled into a very readable reference text with little overlap and a consistent style.

The text is logically broken up into sections on benign disease, clinical evaluation and diagnosis, pathology, screening, treatment and rehabilitation. I found the sections on pathology and aetiology particularly helpful, as were those chapters dealing with benign breast diseases, breast pain and pregnancy. I was also pleased to see that, in addition to the usual general chapters on the management of advanced breast cancer, chapters focusing on site-specific problems were included rather than the typical simplistic approach of considering advanced breast cancer as a single entity. An interesting section is included on rehabilitation, psychosocial issues and quality of life which, while rather targeted to an American readership, discussed many useful general issues.

Inevitably in a text of this size, production takes many months, and as a result the latest references are from 1994. For many sections this does not matter, but clearly in some areas, notably the genetics of familial breast cancer, the role of adjuvant systemic therapy in node-negative patients and new endocrine and cytotoxic agents, there has been considerable progress over the past 2 years.

Reference texts are used for two purposes: firstly, to look up information and, secondly, to obtain guidance on clinical management. I feel that the second area was not given sufficient attention, and there were very few recommendations or judgements made on the clinical use of the multiple treatment approaches now available for both early and advanced breast cancer. Of note, despite the large literature on the subject, there were no recommendations that I could find on appropriate staging investigations. A schema or flow diagram illustrating clinical management decisions would have been a useful addition to each of the relevant chapters on diagnosis, evaluation and treatment.

Notwithstanding all this, I found the book extremely useful. The editors are to be congratulated on the layout and consistency of the text, and I would certainly recommend *Diseases of the Breast* as a reference text for every Breast Unit and Oncology Centre.

*RE Coleman*